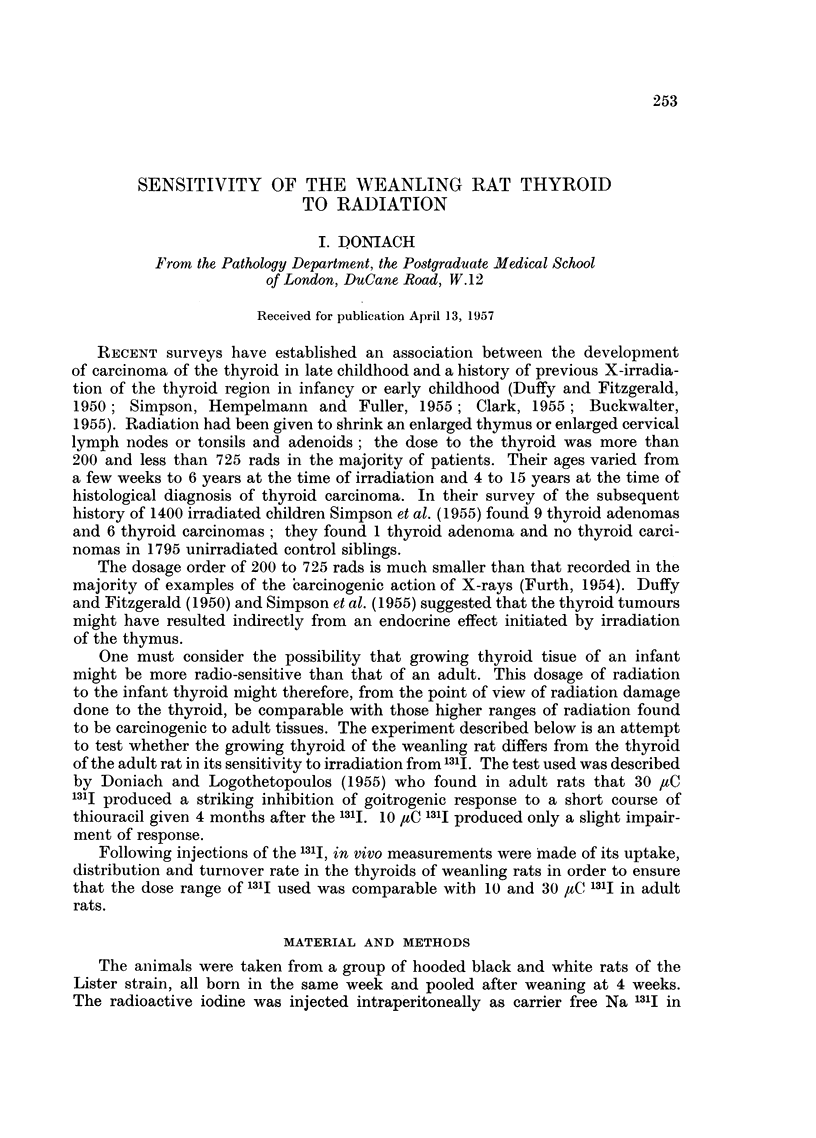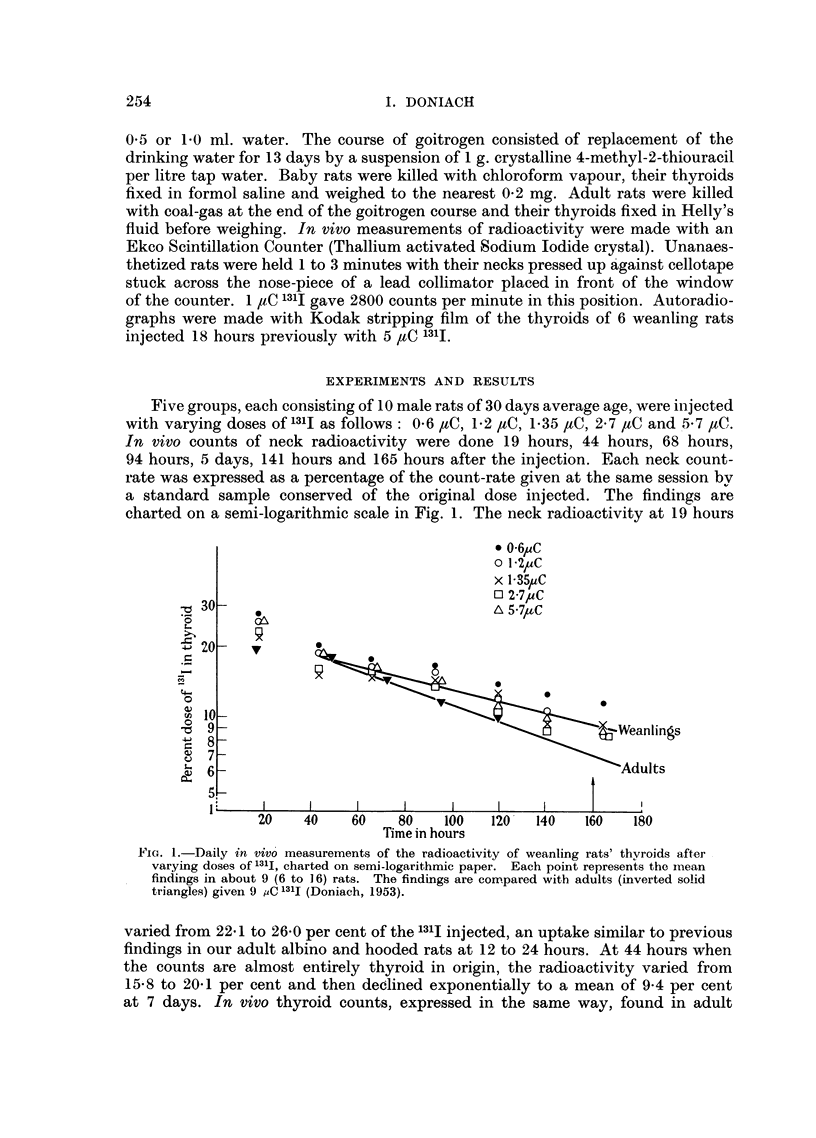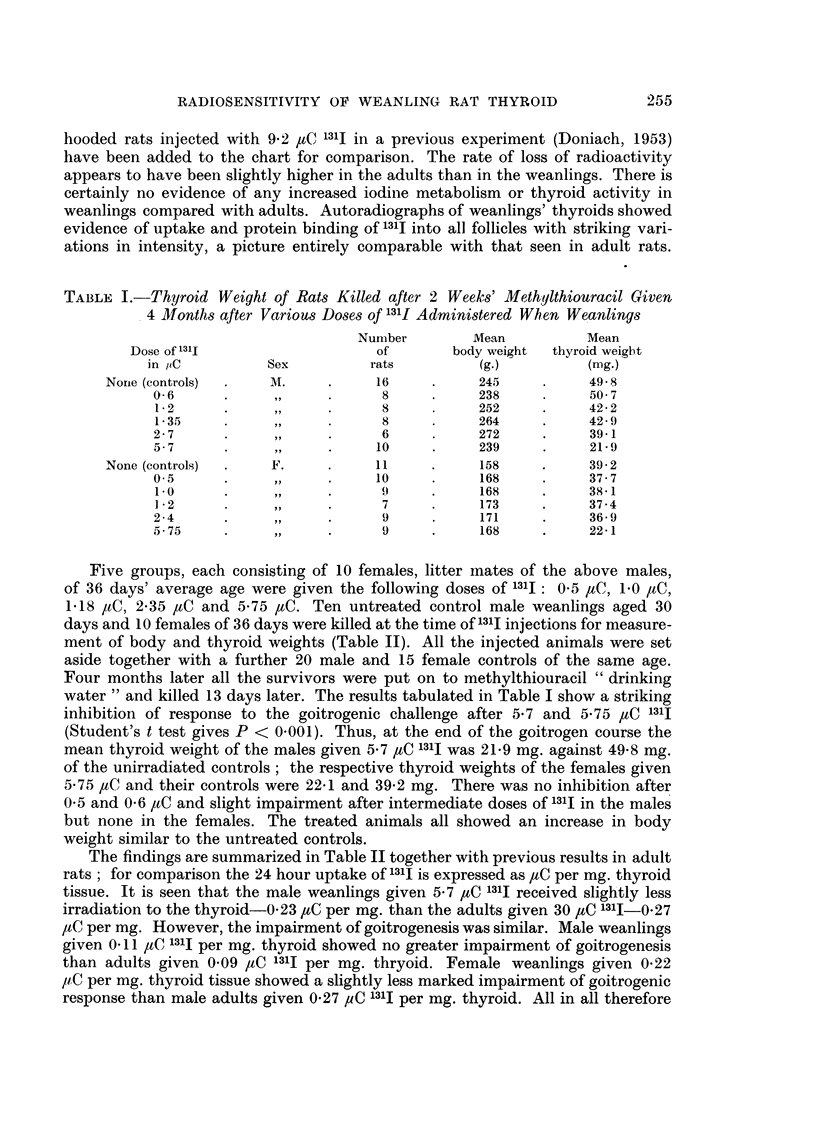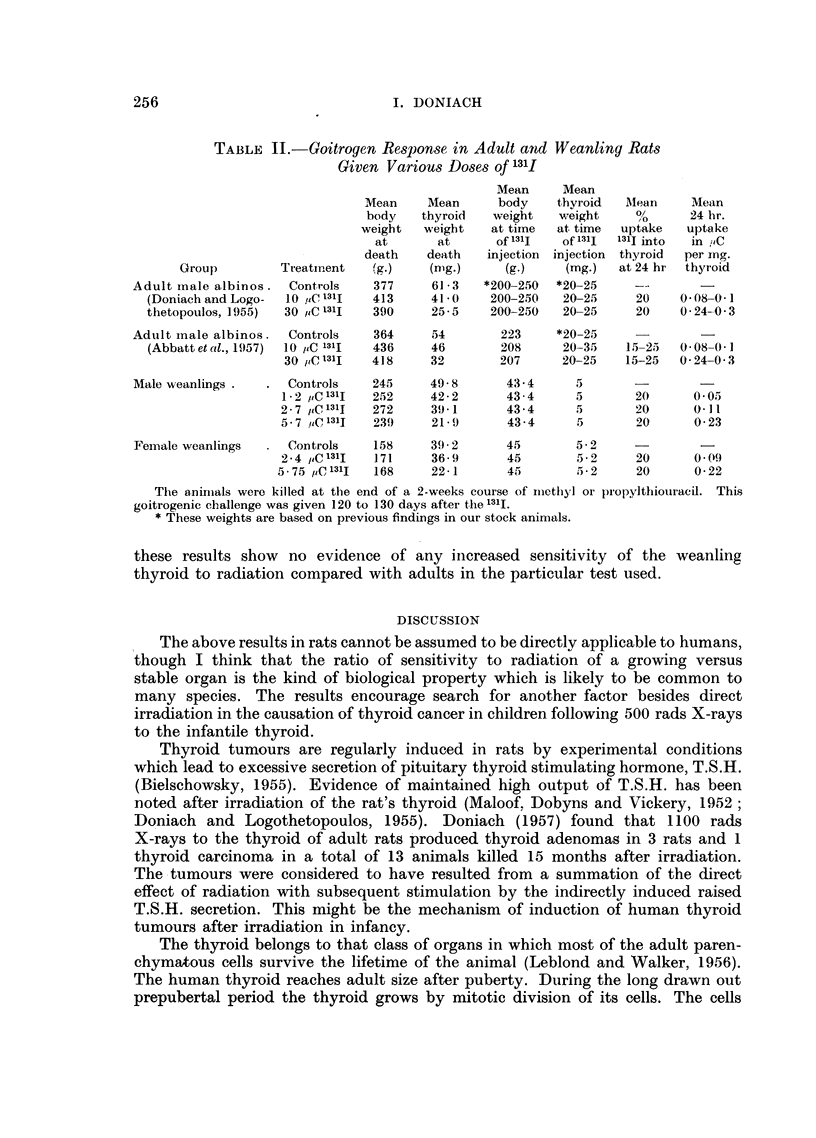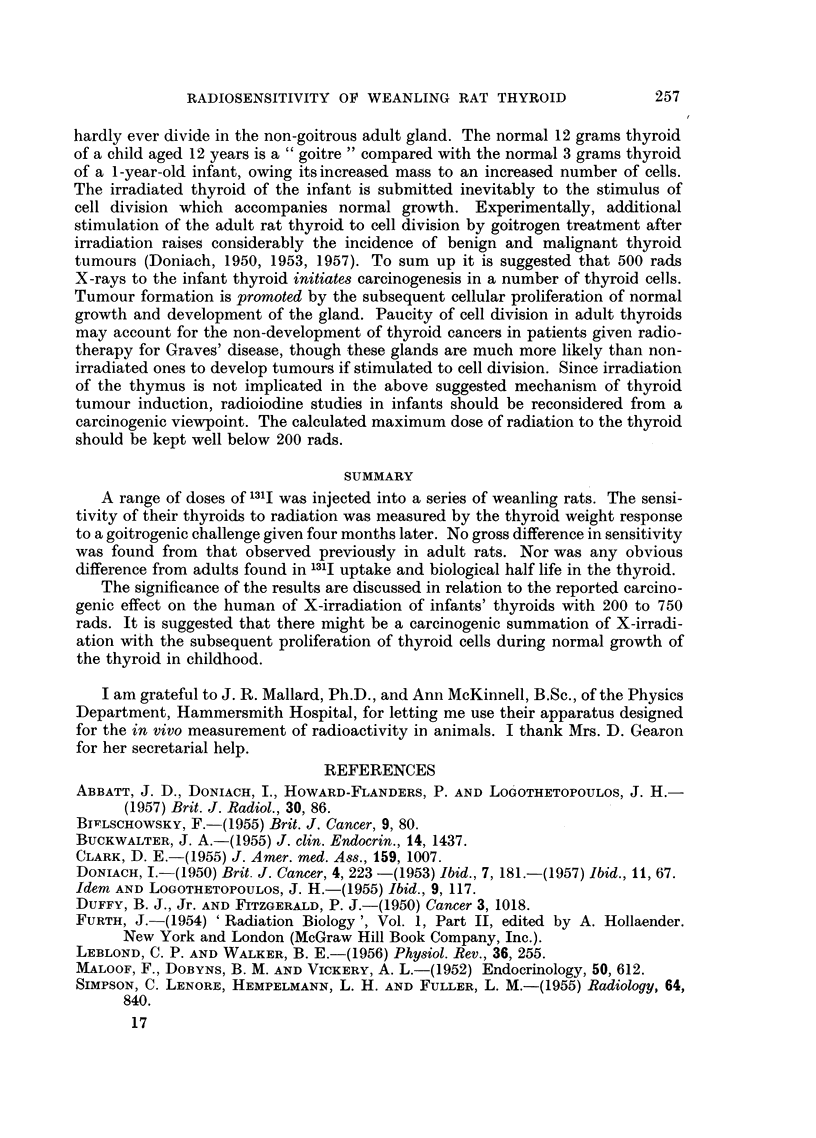# Sensitivity of the Weanling Rat Thyroid to Radiation

**DOI:** 10.1038/bjc.1957.32

**Published:** 1957-06

**Authors:** I. Doniach


### SENSITIVITY OF THE WEANLING RAT THYROID TO RADIATION

I. DONIACH

*From the Pathology Department, the Postgraduate Medical School of London, DuCane Road, W.12*

Received for publication April 13, 1957

RECENT surveys have established an association between the development of carcinoma of the thyroid in late childhood and a history of previous X-irradiation of the thyroid region in infancy or early childhood (Duffy and Fitzgerald, 1950; Simpson, Hempelmann and Fuller, 1955; Clark, 1955; Buckwalter, 1955). Radiation had been given to shrink an enlarged thymus or enlarged cervical lymph nodes or tonsils and adenoids; the dose to the thyroid was more than 200 and less than 725 rads in the majority of patients. Their ages varied from a few weeks to 6 years at the time of irradiation and 4 to 15 years at the time of histological diagnosis of thyroid carcinoma. In their survey of the subsequent history of 1400 irradiated children Simpson *et al.* (1955) found 9 thyroid adenomas and 6 thyroid carcinomas; they found 1 thyroid adenoma and no thyroid carcinomas in 1795 unirradiated control siblings.

The dosage order of 200 to 725 rads is much smaller than that recorded in the majority of examples of the carcinogenic action of X-rays (Furth, 1954). Duffy and Fitzgerald (1950) and Simpson *et al.* (1955) suggested that the thyroid tumours might have resulted indirectly from an endocrine effect initiated by irradiation of the thymus.

One must consider the possibility that growing thyroid tisue of an infant might be more radio-sensitive than that of an adult. This dosage of radiation to the infant thyroid might therefore, from the point of view of radiation damage done to the thyroid, be comparable with those higher ranges of radiation found to be carcinogenic to adult tissues. The experiment described below is an attempt to test whether the growing thyroid of the weanling rat differs from the thyroid of the adult rat in its sensitivity to irradiation from $^{131}I$. The test used was described by Doniach and Logothetopoulos (1955) who found in adult rats that 30 $\mu C$ $^{131}I$ produced a striking inhibition of goitrogenic response to a short course of thiouracil given 4 months after the $^{131}I$. 10 $\mu C$ $^{131}I$ produced only a slight impairment of response.

Following injections of the $^{131}I$, *in vivo* measurements were made of its uptake, distribution and turnover rate in the thyroids of weanling rats in order to ensure that the dose range of $^{131}I$ used was comparable with 10 and 30 $\mu C$ $^{131}I$ in adult rats.

### MATERIAL AND METHODS

The animals were taken from a group of hooded black and white rats of the Lister strain, all born in the same week and pooled after weaning at 4 weeks. The radioactive iodine was injected intraperitoneally as carrier free Na $^{131}I$ in

0·5 or 1·0 ml. water. The course of goitrogen consisted of replacement of the drinking water for 13 days by a suspension of 1 g. crystalline 4-methyl-2-thiouracil per litre tap water. Baby rats were killed with chloroform vapour, their thyroids fixed in formol saline and weighed to the nearest 0·2 mg. Adult rats were killed with coal-gas at the end of the goitrogen course and their thyroids fixed in Helly's fluid before weighing. *In vivo* measurements of radioactivity were made with an Ekco Scintillation Counter (Thallium activated Sodium Iodide crystal). Unanaesthetized rats were held 1 to 3 minutes with their necks pressed up against cellotape stuck across the nose-piece of a lead collimator placed in front of the window of the counter. 1 $\mu$C $^{131}$I gave 2800 counts per minute in this position. Autoradiographs were made with Kodak stripping film of the thyroids of 6 weanling rats injected 18 hours previously with 5 $\mu$C $^{131}$I.

### EXPERIMENTS AND RESULTS

Five groups, each consisting of 10 male rats of 30 days average age, were injected with varying doses of $^{131}$I as follows : 0·6 $\mu$C, 1·2 $\mu$C, 1·35 $\mu$C, 2·7 $\mu$C and 5·7 $\mu$C. *In vivo* counts of neck radioactivity were done 19 hours, 44 hours, 68 hours, 94 hours, 5 days, 141 hours and 165 hours after the injection. Each neck count-rate was expressed as a percentage of the count-rate given at the same session by a standard sample conserved of the original dose injected. The findings are charted on a semi-logarithmic scale in Fig. 1. The neck radioactivity at 19 hours

FIG. 1.—Daily *in vivo* measurements of the radioactivity of weanling rats' thyroids after varying doses of $^{131}$I, charted on semi-logarithmic paper. Each point represents the mean findings in about 9 (6 to 16) rats. The findings are compared with adults (inverted solid triangles) given 9 $\mu$C $^{131}$I (Doniach, 1953).

varied from 22·1 to 26·0 per cent of the $^{131}$I injected, an uptake similar to previous findings in our adult albino and hooded rats at 12 to 24 hours. At 44 hours when the counts are almost entirely thyroid in origin, the radioactivity varied from 15·8 to 20·1 per cent and then declined exponentially to a mean of 9·4 per cent at 7 days. *In vivo* thyroid counts, expressed in the same way, found in adult hooded rats injected with 9·2 $\mu$C $^{131}$I in a previous experiment (Doniach, 1953) have been added to the chart for comparison. The rate of loss of radioactivity appears to have been slightly higher in the adults than in the weanlings. There is certainly no evidence of any increased iodine metabolism or thyroid activity in weanlings compared with adults. Autoradiographs of weanlings' thyroids showed evidence of uptake and protein binding of $^{131}$I into all follicles with striking variations in intensity, a picture entirely comparable with that seen in adult rats.

TABLE I.—*Thyroid Weight of Rats Killed after 2 Weeks' Methylthiouracil Given 4 Months after Various Doses of $^{131}$I Administered When Weanlings*

| Dose of $^{131}$I in $\mu$C | Sex | Number of rats | Mean body weight (g.) | Mean thyroid weight (mg.) |
|---|---|---|---|---|
| None (controls) | M. | 16 | 245 | 49·8 |
| 0·6 | ,, | 8 | 238 | 50·7 |
| 1·2 | ,, | 8 | 252 | 42·2 |
| 1·35 | ,, | 8 | 264 | 42·9 |
| 2·7 | ,, | 6 | 272 | 39·1 |
| 5·7 | ,, | 10 | 239 | 21·9 |
| None (controls) | F. | 11 | 158 | 39·2 |
| 0·5 | ,, | 10 | 168 | 37·7 |
| 1·0 | ,, | 9 | 168 | 38·1 |
| 1·2 | ,, | 7 | 173 | 37·4 |
| 2·4 | ,, | 9 | 171 | 36·9 |
| 5·75 | ,, | 9 | 168 | 22·1 |

Five groups, each consisting of 10 females, litter mates of the above males, of 36 days' average age were given the following doses of $^{131}$I : 0·5 $\mu$C, 1·0 $\mu$C, 1·18 $\mu$C, 2·35 $\mu$C and 5·75 $\mu$C. Ten untreated control male weanlings aged 30 days and 10 females of 36 days were killed at the time of $^{131}$I injections for measurement of body and thyroid weights (Table II). All the injected animals were set aside together with a further 20 male and 15 female controls of the same age. Four months later all the survivors were put on to methylthiouracil " drinking water " and killed 13 days later. The results tabulated in Table I show a striking inhibition of response to the goitrogenic challenge after 5·7 and 5·75 $\mu$C $^{131}$I (Student's $t$ test gives $P < 0{\cdot}001$). Thus, at the end of the goitrogen course the mean thyroid weight of the males given 5·7 $\mu$C $^{131}$I was 21·9 mg. against 49·8 mg. of the unirradiated controls ; the respective thyroid weights of the females given 5·75 $\mu$C and their controls were 22·1 and 39·2 mg. There was no inhibition after 0·5 and 0·6 $\mu$C and slight impairment after intermediate doses of $^{131}$I in the males but none in the females. The treated animals all showed an increase in body weight similar to the untreated controls.

The findings are summarized in Table II together with previous results in adult rats ; for comparison the 24 hour uptake of $^{131}$I is expressed as $\mu$C per mg. thyroid tissue. It is seen that the male weanlings given 5·7 $\mu$C $^{131}$I received slightly less irradiation to the thyroid—0·23 $\mu$C per mg. than the adults given 30 $\mu$C $^{131}$I—0·27 $\mu$C per mg. However, the impairment of goitrogenesis was similar. Male weanlings given 0·11 $\mu$C $^{131}$I per mg. thyroid showed no greater impairment of goitrogenesis than adults given 0·09 $\mu$C $^{131}$I per mg. thryoid. Female weanlings given 0·22 $\mu$C per mg. thyroid tissue showed a slightly less marked impairment of goitrogenic response than male adults given 0·27 $\mu$C $^{131}$I per mg. thyroid. All in all therefore

TABLE II.—*Goitrogen Response in Adult and Weanling Rats Given Various Doses of* $^{131}I$

| Group | Treatment | Mean body weight at death (g.) | Mean thyroid weight at death (mg.) | Mean body weight at time of $^{131}$I injection (g.) | Mean thyroid weight at time of $^{131}$I injection (mg.) | Mean % uptake $^{131}$I into thyroid at 24 hr | Mean 24 hr. uptake in $\mu$C per mg. thyroid |
|---|---|---|---|---|---|---|---|
| Adult male albinos . | Controls | 377 | 61·3 | *200–250 | *20–25 | — | — |
| (Doniach and Logo- | 10 $\mu$C $^{131}$I | 413 | 41·0 | 200–250 | 20–25 | 20 | 0·08–0·1 |
| thetopoulos, 1955) | 30 $\mu$C $^{131}$I | 390 | 25·5 | 200–250 | 20–25 | 20 | 0·24–0·3 |
| Adult male albinos . | Controls | 364 | 54 | 223 | *20–25 | — | — |
| (Abbatt *et al.*, 1957) | 10 $\mu$C $^{131}$I | 436 | 46 | 208 | 20–35 | 15–25 | 0·08–0·1 |
| | 30 $\mu$C $^{131}$I | 418 | 32 | 207 | 20–25 | 15–25 | 0·24–0·3 |
| Male weanlings . . | Controls | 245 | 49·8 | 43·4 | 5 | — | — |
| | 1·2 $\mu$C $^{131}$I | 252 | 42·2 | 43·4 | 5 | 20 | 0·05 |
| | 2·7 $\mu$C $^{131}$I | 272 | 39·1 | 43·4 | 5 | 20 | 0·11 |
| | 5·7 $\mu$C $^{131}$I | 239 | 21·9 | 43·4 | 5 | 20 | 0·23 |
| Female weanlings . | Controls | 158 | 39·2 | 45 | 5·2 | — | — |
| | 2·4 $\mu$C $^{131}$I | 171 | 36·9 | 45 | 5·2 | 20 | 0·09 |
| | 5·75 $\mu$C $^{131}$I | 168 | 22·1 | 45 | 5·2 | 20 | 0·22 |

The animals were killed at the end of a 2-weeks course of methyl or propylthiouracil. This goitrogenic challenge was given 120 to 130 days after the $^{131}$I.

* These weights are based on previous findings in our stock animals.

these results show no evidence of any increased sensitivity of the weanling thyroid to radiation compared with adults in the particular test used.

### DISCUSSION

The above results in rats cannot be assumed to be directly applicable to humans, though I think that the ratio of sensitivity to radiation of a growing versus stable organ is the kind of biological property which is likely to be common to many species. The results encourage search for another factor besides direct irradiation in the causation of thyroid cancer in children following 500 rads X-rays to the infantile thyroid.

Thyroid tumours are regularly induced in rats by experimental conditions which lead to excessive secretion of pituitary thyroid stimulating hormone, T.S.H. (Bielschowsky, 1955). Evidence of maintained high output of T.S.H. has been noted after irradiation of the rat's thyroid (Maloof, Dobyns and Vickery, 1952; Doniach and Logothetopoulos, 1955). Doniach (1957) found that 1100 rads X-rays to the thyroid of adult rats produced thyroid adenomas in 3 rats and 1 thyroid carcinoma in a total of 13 animals killed 15 months after irradiation. The tumours were considered to have resulted from a summation of the direct effect of radiation with subsequent stimulation by the indirectly induced raised T.S.H. secretion. This might be the mechanism of induction of human thyroid tumours after irradiation in infancy.

The thyroid belongs to that class of organs in which most of the adult parenchymatous cells survive the lifetime of the animal (Leblond and Walker, 1956). The human thyroid reaches adult size after puberty. During the long drawn out prepubertal period the thyroid grows by mitotic division of its cells. The cells hardly ever divide in the non-goitrous adult gland. The normal 12 grams thyroid of a child aged 12 years is a " goitre " compared with the normal 3 grams thyroid of a 1-year-old infant, owing its increased mass to an increased number of cells. The irradiated thyroid of the infant is submitted inevitably to the stimulus of cell division which accompanies normal growth. Experimentally, additional stimulation of the adult rat thyroid to cell division by goitrogen treatment after irradiation raises considerably the incidence of benign and malignant thyroid tumours (Doniach, 1950, 1953, 1957). To sum up it is suggested that 500 rads X-rays to the infant thyroid *initiates* carcinogenesis in a number of thyroid cells. Tumour formation is *promoted* by the subsequent cellular proliferation of normal growth and development of the gland. Paucity of cell division in adult thyroids may account for the non-development of thyroid cancers in patients given radiotherapy for Graves' disease, though these glands are much more likely than non-irradiated ones to develop tumours if stimulated to cell division. Since irradiation of the thymus is not implicated in the above suggested mechanism of thyroid tumour induction, radioiodine studies in infants should be reconsidered from a carcinogenic viewpoint. The calculated maximum dose of radiation to the thyroid should be kept well below 200 rads.

SUMMARY

A range of doses of $^{131}I$ was injected into a series of weanling rats. The sensitivity of their thyroids to radiation was measured by the thyroid weight response to a goitrogenic challenge given four months later. No gross difference in sensitivity was found from that observed previously in adult rats. Nor was any obvious difference from adults found in $^{131}I$ uptake and biological half life in the thyroid.

The significance of the results are discussed in relation to the reported carcinogenic effect on the human of X-irradiation of infants' thyroids with 200 to 750 rads. It is suggested that there might be a carcinogenic summation of X-irradiation with the subsequent proliferation of thyroid cells during normal growth of the thyroid in childhood.

I am grateful to J. R. Mallard, Ph.D., and Ann McKinnell, B.Sc., of the Physics Department, Hammersmith Hospital, for letting me use their apparatus designed for the *in vivo* measurement of radioactivity in animals. I thank Mrs. D. Gearon for her secretarial help.

### REFERENCES

ABBATT, J. D., DONIACH, I., HOWARD-FLANDERS, P. AND LOGOTHETOPOULOS, J. H.—(1957) *Brit. J. Radiol.*, **30**, 86.
BIELSCHOWSKY, F.—(1955) *Brit. J. Cancer*, **9**, 80.
BUCKWALTER, J. A.—(1955) *J. clin. Endocrin.*, **14**, 1437.
CLARK, D. E.—(1955) *J. Amer. med. Ass.*, **159**, 1007.
DONIACH, I.—(1950) *Brit. J. Cancer*, **4**, 223 —(1953) *Ibid.*, **7**, 181.—(1957) *Ibid.*, **11**, 67.
*Idem* AND LOGOTHETOPOULOS, J. H.—(1955) *Ibid.*, **9**, 117.
DUFFY, B. J., Jr. AND FITZGERALD, P. J.—(1950) *Cancer* **3**, 1018.
FURTH, J.—(1954) ' Radiation Biology ', Vol. 1, Part II, edited by A. Hollaender. New York and London (McGraw Hill Book Company, Inc.).
LEBLOND, C. P. AND WALKER, B. E.—(1956) *Physiol. Rev.*, **36**, 255.
MALOOF, F., DOBYNS, B. M. AND VICKERY, A. L.—(1952) Endocrinology, **50**, 612.
SIMPSON, C. LENORE, HEMPELMANN, L. H. AND FULLER, L. M.—(1955) *Radiology*, **64**, 840.